# Expression of a Highly Antigenic and Native-Like Folded Extracellular Domain of the Human α1 Subunit of Muscle Nicotinic Acetylcholine Receptor, Suitable for Use in Antigen Specific Therapies for Myasthenia Gravis

**DOI:** 10.1371/journal.pone.0084791

**Published:** 2013-12-20

**Authors:** Athanasios Niarchos, Marios Zouridakis, Vassilis Douris, Assimina Georgostathi, Dimitra Kalamida, Alexandros Sotiriadis, Konstantinos Poulas, Kostas Iatrou, Socrates J. Tzartos

**Affiliations:** 1 Department of Pharmacy, University of Patras, Patras, Greece; 2 Department of Biochemistry, Hellenic Pasteur Institute, Athens, Greece; 3 Institute of Biosciences and Applications, National Centre for Scientific Research “Demokritos”, Athens, Greece; University of Sydney, Australia

## Abstract

We describe the expression of the extracellular domain of the human α1 nicotinic acetylcholine receptor (nAChR) in lepidopteran insect cells (i-α1-ECD) and its suitability for use in antigen-specific therapies for Myasthenia Gravis (MG). Compared to the previously expressed protein in *P. pastoris* (y-α1-ECD), i**-**α1**-**ECD had a 2-fold increased expression yield, bound anti-nAChR monoclonal antibodies and autoantibodies from MG patients two to several-fold more efficiently and resulted in a secondary structure closer to that of the crystal structure of mouse α1-ECD. Our results indicate that i**-**α1**-**ECD is an improved protein for use in antigen-specific MG therapeutic strategies.

## Introduction

Chemical transmission of electrical signals from the nervous system to the skeletal muscles is mediated by the muscle nicotinic acetylcholine receptor (nAChR) at the neuromuscular junction [1]. The muscle nAChR is a transmembrane glycoprotein [Molecular mass (*M*r) ~290 kDa], consisting of five homologous subunits forming an ionic channel with stoichiometry (α1)_2_β1γδ in embryos or (α1)_2_β1εδ in adults. Muscle nAChR is also the major target of the autoantibodies in most myasthenia gravis (MG) patients [2].

In order to bind the majority of MG autoantibodies and remove them from MG patients’ sera, the most appropriate antigen to be used for immunoadsorption columns preparation is the extracellular domain (ECD) of the α subunit of the muscle-type nAChR (α1-ECD). α1-ECD not only contains the main immunogenic region (MIR), which is a major target of the anti-nAChR autoantibodies in MG [3], but also is water soluble [4] and expressed in a much higher yield than the transmembrane, water-insoluble, full-length subunit. For this purpose, in previous studies, human α1-ECD (amino acids 1–210) has been tested for binding of MG autoantibodies in immunoadsorption columns, after expression in *E. coli* [5] and in the yeast *P. pastoris* [6].

In the present study, the human α1-ECD was expressed in stably transformed lepidopteran insect cells (i-α1-ECD); an expression system particularly suited for production of soluble proteins [7]. This system has been successfully used for expression of several proteins of mammalian origin, since it has the advantage of conferring posttranslational modifications very similar to the mammalian and of allowing relatively high levels of protein expression [7-9]. i-α1-ECD was expressed as a water-soluble monomeric molecule in sufficient quantities for future clinical use. The i-α1-ECD was found to bind more efficiently anti-nAChR monoclonal antibodies (mAbs), as well as autoantibodies from MG patients compared to the homologous protein previously expressed in *P. pastoris* (y-α1-ECD). Together with far-UV CD studies, i-α1-ECD was proved to acquire a more native conformation and antigenicity compared to y-α1-ECD, and therefore it would be more appropriate for use as a tool for the development of therapeutic approaches in MG.

## Materials and Methods

### Human α1 nAChR ECD expression in High-Five^TM^ insect cells and in the yeast *P. pastoris*

For expression in insect cells, a 708 bp fragment of the human α1-ECD cDNA, encoding the first 231 amino acids starting from the N-terminus, was cloned into the BamHI restriction site of plasmid pEIA.PXaMycHis [7]. The recombinant fragment was led by the native signal peptide for secretion (20 a/a) whereas at the C-terminal end, it was fused to a sequence encoding the c-myc epitope and polyhistidine (6xHis) tag, preceded by a factor Xa proteolytic domain. *Trichoplusia ni* BTI-TN-5B1-4 cells (HighFive^TM^; Invitrogen) cultured in ESF921 serum-free medium (Protein Expression Systems) were transfected with Lipofectin^TM^ reagent (Invitrogen) [10] and the stably transfected cell lines were selected as previously described [7]. Static cultures were seeded at a density of 2x10^5^ cells/mL, while suspension cultures started from the density of 1x10^6^ cells/mL, in Express Five medium (Invitrogen) supplemented with L-glutamine (16 mM), puromycin (15 μg/mL) and gentamycin (50 μg/mL) at 27 °C. When the culture reached a cell density of 5x10^6^ cells/mL, it was centrifuged at 600 g for 20 min at 4 °C and the supernatant was harvested for protein purification. For expression in *P. pastoris*, cDNA was cloned into the pPICZαA vector (Invitrogen) and the protein was expressed in the GS115 strain of *P. pastoris*, as previously described [4].

### Protein purification

After filtration through 0.2 μm filter, the supernatant of either *High Five* or *P. pastoris* culture was concentrated using a 10 kDa ultrafiltration system (Ultrasette, Pall Corporation) and dialyzed against 50 mM phosphate buffer (PB), 500 mM NaCl, 10 mM imidazole, pH 8.0, and the protein was purified using Ni-NTA metal affinity chromatography (Qiagen), based on the C-terminal 6×His tag. The washing and elution steps were performed at 40 mM and 150 mM imidazole concentrations, respectively. The ECDs were further purified by gel filtration using a Superose-12 column (Amersham Biosciences) on a FPLC system (AKTApurifier-10) using phosphate-buffered saline (PBS), pH 7.4, at a flow rate of 0.5 mL/min. Fraction volumes were set at 0.5 mL. Protein concentration was determined using the Bradford assay method.

### Circular dichroism studies

Far-UV circular dichroism (CD) spectra were recorded at 20 °C using a Jasco J-715 spectropolarimeter (Japan Spectroscopic Co.). The scan speed was set at 50 nm/min, the bandwidth at 1 nm, the response time at 2 s, the upper limit of the High Tension voltage at 600 V, the scan ranges at 190–260 nm (far-UV) and the resolution at 0.2 nm. The quartz cell path length was 1 mm and the purified i-α1-ECD (0.3 mg/mL) was dissolved in 10 mM PB, 50 mM sodium fluoride, pH 7.5. The derived spectrum represents the average of ten scans after subtraction of a buffer blank. The secondary structure composition was calculated using the programs CDSSTR, CONTINLL and SELCON3 included in the CDPro software [11].

### Dynamic light scattering (DLS) analysis

DLS analysis of purified i-α1-ECD was performed using a Zetasizer NanoS Instrument (Malvern Instruments, UK) and the results were analyzed with DTS v.4.1 software.

### SDS-PAGE and western blot analysis

The purified ECDs were analyzed by 12% SDS-PAGE followed by Coomassie staining. For Western blot analysis the protein bands were transferred on a nitrocellulose membrane (Amersham Biosciences), and after blocking in PBS, 5% milk, were probed with the anti-α1 mAb 198 [12] (1:1,000 in PBS, pH 7.4, 0.2% BSA) and subsequently with rabbit anti-rat peroxidase conjugated immunoglobulins (Pierce, dilution 1:1,000 in PBS, pH 7.4, 0.2% BSA). Finally, the bands were observed by incubation in DAB (3,3'-diaminobenzidine) staining buffer (PBS, 0.5 mg/mL DAB, 2 mM NiCl_2_, 0.02% H_2_O_2_).

### Filter Assay for ^125^I-α-BTX binding to α1-ECD

Up to 400 ng of purified α1-ECDs were incubated at 4 °C for 3 h with 50,000 cpm of ^125^I-α-BTX in a final volume of 50 μL of PBS, 0.2% BSA, pH 7.4. The samples were then diluted with 1 mL of 0.5% Triton X-100 in 20 mM Tris buffer, pH 7.4 (Triton buffer) and immediately filtered through Whatman DE81 anion-exchange filters pre-soaked with Triton buffer. The filters were then washed twice with 1 mL of Triton buffer, the bound radioactivity was measured, and after subtracting the non-specific bound radioactivity, using samples without the α1-ECD, it was expressed as the specifically bound radioactivity (Δcpm). Unpaired t-tests were performed between the bound Δcpm values, scored by i-α1-ECD and y-α1-ECD, for every amount used of each α1-ECD.

### Ligand competition experiments

Specific binding of small nicotinic ligands such as nicotine, carbamylcholine, d-tubocurarine and gallamine was studied in competition experiments with ^125^I-α-BTX. Various concentrations of the unlabeled ligands, or NaCl as negative control, were added simultaneously with 50,000 cpm of radio-labeled α-bungarotoxin (^125^I-α-BTX) to 20 ng of α1-ECD in a final volume of 50 μL in PBS buffer, 0.2% BSA, pH 7.4, and the mixture was incubated at 4 °C for 3 h. Bound radioactivity was then measured as above. The residual ^125^I-α-BTX binding ability was determined as the ratio (%) of the specific radioactivity (Δcpm) bound in the presence and absence of the unlabeled ligand. 

### ELISA for mAbs

Wells of microtiter plates (MaxiSorp F96, Nunc) were coated with 40 ng of α1-ECD, and after blocking with PBS, 2% BSA, pH 7.4, 100 μL of various dilutions of mAbs (in PBS pH 7.4, 0.2% BSA) were added to each well, or 10 μL of normal rat serum as the negative control, and the plates were incubated for 3 h at RT. Subsequently 100 μL of peroxidase-conjugated rabbit anti-rat IgG (Pierce, dilution 1:500 in PBS, pH 7.4, 0.2% BSA) were added to each well and incubated for 2 h at RT. Bound mAbs were determined colorimetrically using TMB (3,3´, 5,5´-tetramethylbenzidine) as substrate. The developed color was measured by a microtiter plate reader at 450 nm after addition of 1 M H_2_SO_4_. Unpaired t-tests were performed between the A_450_ values, scored by i-α1-ECD and y-α1-ECD, for every volume used of each mAb.

### Radioimmunoassay (RIA) for MG patients’ sera

40 ng of α1-ECD were labeled with ^125^I-α-BTX (50,000 cpm) in a total volume of 45 μL of PBS, 0.2% BSA, pH 7.4, for 3 h at 4 °C. After addition of 1-5 μL of MG patients’ sera or normal human serum (NHS) as negative control and PBS, 0.2% BSA, pH 7.4 up to a total volume of 50 μL, the samples were incubated at 4 °C for 15–18 h, and then the immune complexes were precipitated by addition of 25 μL of goat anti-human immunoglobulin antiserum and incubation for 1.5 h at 4 °C. Subsequently, 1 mL of PBS buffer was added, followed by centrifugation (6,000 g, 4 °C, 10 min). The precipitates were washed twice with 1 mL PBS buffer and the precipitated radioactivity was counted. After subtracting the background cpm when using NHS, the precipitated radioactivity was expressed as the ratio (%) of the Δcpm counted in the RIA to the Δcpm counted in the filter assay, when using with the same amount of α1-ECD (40 ng) and ^125^I-α-BTX (50,000 cpm). Unpaired t-tests were performed between the % bound precipitated radioactivity values, scored by i-α1-ECD and y-α1-ECD, for every volume used of each serum.

## Results

### Gel filtration, far-UV CD analysis and dynamic light scattering analysis of i-α1-ECD

**Figure 1:**
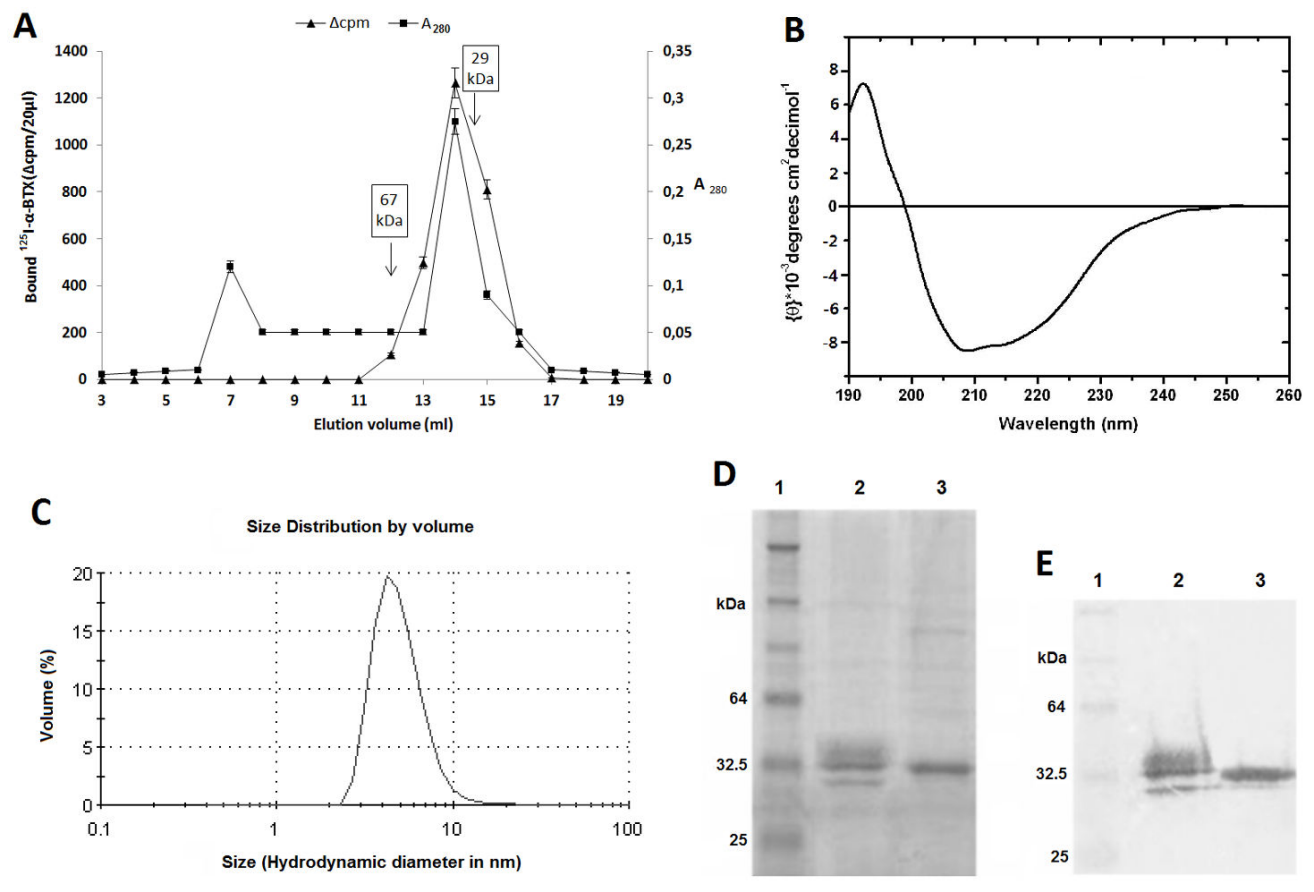
Biochemical and biophysical analysis of i-α1-ECD. **A**) **Gel filtration analysis**. The right y axis shows the absorbance at 280 nm (A_280_) and the left y axis the bound ^125^I-α-BTX (Δcpm) in the filter assay, using 20 μL of each gel filtration fraction and 3,000 cpm of ^125^I-α-BTX. The values are the mean and standard deviation (±S.D.). Arrows indicate the peaks of eluted protein markers of known molecular mass. **B**) **Far-UV CD analysis**. Deconvolution of the presented spectrum with CDPro software showed 8% α-helical content, 40% β-sheet structure, 22% β-turns and 30% unordered portion. NRMSD (normalized root mean square deviation) value was 0.05, reflecting the accurate analysis of secondary structure. **C**) **DLS analysis**. The size distribution by volume of the gel-filtration isolated i-α1-ECD molecules is shown. The calculated hydrodynamic diameter of i-α1-ECD is 4 nm with a polydispersity value of 29%. Protein sample was derived from gel filtration analysis and its concentration was 0.3 mg/mL. SDS-PAGE analysis followed by D) Coomassie Brilliant Blue staining and **E**) Western blot analysis using the anti-α1 nAChR mAb 198. Lane 1: Positions of protein Mr standards; Lane 2: y-α1-ECD; Lane 3: i-α1-ECD. Protein samples were derived from gel filtration chromatography.

The solubility and size of i-α1-ECD were studied by gel filtration, following its first step Ni-NTA metal affinity-based purification, and DLS analysis. As shown in Figure **1A**, ^125^I-α-BTX binding revealed that i-α1-ECD was expressed exclusively in a monomeric form (Mr ~32 kDa), similarly to the previously studied y-α1-ECD [4]. The first A_280_ peak of the chromatograph is attributed to a high molecular mass contaminant, as determined by SDS-PAGE (data not shown). The expression yield of the finally purified i-α1-ECD was estimated by the Bradford assay to be ~0.5 mg/L, thus being 2-fold higher than that described for the y-α1-ECD [4]. The far-UV CD spectrum of i-α1-ECD was characteristic of a β-rich protein, as this presented a positive Cotton effect in the 190–200 nm region and a negative one in the 200–220 nm region (Figure **1B**), suggesting a major contribution from β-sheet structure [13]. Deconvolution of the derived spectrum revealed a secondary structure composition of 40% β-sheet structure and 8% α-helical content, while a large proportion of secondary structure is attributed to unordered elements (30%) and to β-turns (22%).

Consistent with gel filtration analysis-based calculated Mr is the DLS analysis (Figure **1C**) for the i-α1-ECD, which showed a hydrodynamic diameter of 4 nm, corresponding to a globular protein of ~30 kDa. Moreover, DLS revealed a significant monodispersity (Calculated polydispersity value of 29%) of the expressed i-α1-ECD molecules, which remained stable in concentrations values of at least 10 mg/mL, a good starting point for crystallization trials.

### SDS-PAGE and western blot analysis of i-α1-ECD and y-α1-ECD

Both Coomassie-blue stained SDS-PAGE (Figure **1D**) and western blot analysis (Figure **1E**), using the anti-α1 mAb 198, indicated that i-α1-ECD has approximately the same Mr with y-α1-ECD (~32 kDa), which is consistent with that deduced from gel filtration and DLS analysis, but higher than the theoretically predicted based on its primary sequence, probably attributed to glycosylation. Interestingly, i-α1-ECD, in contrast to y-α1-ECD, migrated as a single sharp band, suggesting that its glycosylation is highly homogeneous, an important feature for facilitating crystallization trials for analytical structural studies. 

### Binding of ^125^I-α-BTX and small cholinergic ligands to i-α1-ECD and y-α1-ECD

**Figure 2:**
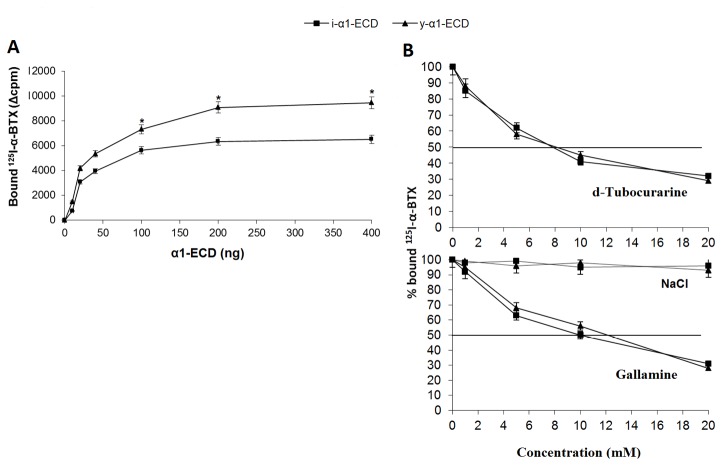
Ligand binding of i-α1-ECD and y-α1-ΕCD. **A**) **^125^I-α-BTX-binding ability**. Up to 400 ng of gel filtration purified y-α1-ECD and i-α1-ECD were incubated with 50,000 cpm of ^125^I-α-BTX in the filter assay and the bound radioactivity measured on a γ-counter. **B**) **Competition between ^125^I-α-BTX and small unlabelled ligands for binding to y-α1-ECD and i-α1-ECD**. Inhibition of ^125^I-α-BTX-binding by d-tubocurarine and gallamine to both α1-ECDs was assessed by co-incubation competition experiments with unlabelled ligands. Binding of ^125^I-α-BTX in the absence of any unlabeled ligand was defined as the 100% of binding. Sodium chloride did not significantly inhibit ligand binding. The values are the mean and standard deviation (±S.D.) from 5 experiments. ^*^Statistically significant differences (unpaired t-test, p<0.05). ^**^Statistically very significant differences (unpaired t-test, p<0.01).

The ability of both i-α1-ECD and y-α1-ECD to bind ^125^I-α-BTX was tested in the filter assay. As shown in Figure **2A**, the ^125^I-α-BTX binding-ability of i-α1-ECD was somewhat lower (by about one third) than that of y-α1-ECD, which has a K_d_ value of 5 nM for ^125^I-α-BTX [4]. To further compare the ligand-binding ability of i-α1-ECD and y-α1-ECD, we then tested the binding of four small cholinergic ligands (d-tubocurarine, nicotine, carbamylcholine and gallamine). Binding of these ligands to both α1-ECDs was assessed indirectly by their ability to inhibit the binding of ^125^I-α-BTX in competition experiments. The small agonists carbamylcholine and nicotine did not affect ^125^I-α-BTX binding to any of the two ECDs, even at concentrations of up to 10 mM (data not shown). However, the two competitive antagonists, gallamine and d-tubocurarine presented IC_50_ values for inhibition of ^125^I-α-BTX binding to both y-α1-ECD and i-α1-ECD at ~8 mM and ~10 mM, respectively, without significant difference between the two ECDs (Figure **2B**).

### Binding of partially and completely conformation-dependent anti-nAChR mAbs to i-α1-ECD and y-α1-ECD

**Figure 3:**
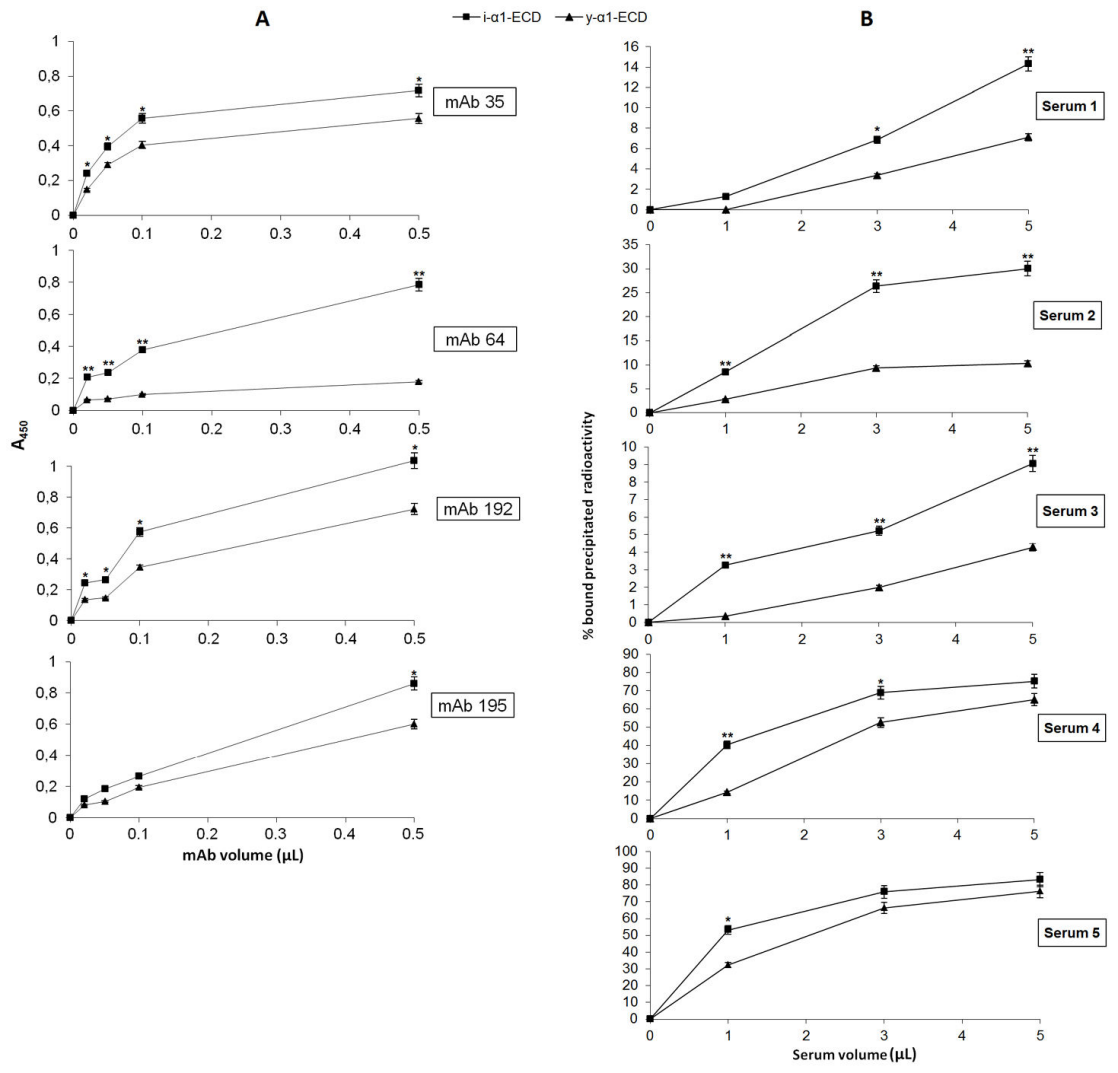
Binding of antibodies to y-α1-ECD and i-α1-ECD. **A**) **Anti-nAChR mAbs binding**. Partially conformation-dependent anti-MIR mAb (mAb 195), strictly conformation-dependent anti-MIR mAbs (mAbs 192 and 35) and mAb 64 (a conformation-dependent anti-α1, non anti-MIR mAb), were tested in ELISA experiments showing the binding of the anti-nAChR mAbs at increasing volumes to 40 ng of y-α1-ECD or i-α1-ECD. **B**) **Binding of anti-α1 autoantibodies from MG sera**. Five anti-nAChR sera were assayed by RIA for binding to 40 ng of purified y-α1-ECD or i-α1-ECD, labeled with 50,000 cpm ^125^I-α-BTX. Sera 1, 2 and 3 have low titers and sera 4 and 5 are of high titer for anti-nAChR autoantibodies. Bound radioactivity to the immunoprecipitates (Y-axis) was expressed as the % ratio to the bound radioactivity when y-α1-ECD or i-α1-ECD filter assayed with 50,000 cpm ^125^I-α-BTX. The values are the mean and standard deviation (±S.D.) from 5 experiments. ^*^Statistically significant differences (unpaired t-test, p<0.05). ^**^Statistically very significant differences (unpaired t-test, p<0.01).

Several anti-nAChR mAbs were tested for binding to both i-α1-ECD and y-α1-ECD, using ELISA (Figure **3A**). mAbs 192, 64 and 35 are known to bind almost exclusively to the native human nAChR, whereas binding of mAb 195 is only partially conformation-dependent [12,14]. It is shown that all mAbs tested, bound at least 2-fold better to i-α1-ECD than y-α1-ECD.

### Binding of autoantibodies from MG patients’ sera to i-α1-ECD and y-α1-ECD

We then proceeded in comparing the binding abilities of i-α1-ECD and y-α1-ECD to anti-nAChR autoantibodies present in five MG patients’ sera, using RIA experiments; three of these sera (sera #1, #2 and #3) have low to moderate titers of anti-nAChR autoantibodies (titers values of 1.9 nM, 16 nM and 15 nM, respectively) and two sera (sera #4, #5) have very high titers (1 and 2 μΜ, respectively). Furthermore, since anti-nAChR autoantibodies are generally highly conformation-dependent and bind to several epitopes on each subunit [15], this set of RIA experiments would also be further indicative of which of both proteins adopts the most appropriate native-like conformation. It was shown that all 5 sera bound much better to i-α1-ECD than to y-α1-ECD (Figure **3B**).

## Discussion

Human α1-ECD, was expressed in stably transformed lepidopteran insect cells in order to obtain a recombinant protein with better immunoadsorption potential for future MG therapies. Gel filtration chromatography (Figure **1A**) and DLS analysis (Figure **2C**) indicated that i-α1-ECD was expressed in the form of water-soluble monomer with a molecular weight (Mr~32 kDa) close to the theoretically predicted based on its primary structure (theoretical Mr~28kDa) and of a satisfactory degree of monodispersity. The difference between the calculated and expected Mr is probably attributed to glycosylation. The expression yield of i-α1-ECD (0.5 mg/L) was 2-fold increased compared to that of y-α1-ECD [4]. Moreover, SDS-PAGE (Figure **1D**) and Western blot analysis (Figure **1E**), apart from confirming the Mr calculated from gel filtration and DLS analysis, also revealed a more homogeneous glycosylation pattern for the i-α1-ECD conferred by the insect cell expression system. As shown, i-α1-ECD migrated as a sharp band, whereas a smear was observed in the case of y-α1-ECD. Yeast-expressed proteins are known to have very long (and heterogeneous) carbohydrate chains [16], very different from the mammalian-expressed proteins [17]. In contrast, the glycosylation pattern of insect-expressed proteins is more closely related to the mammalian [18]. Interestingly, apart from addressing the question of the improved antigenicity of the i- α1-ECD, which is the main purpose of the current study, the relatively high monodispersity and homogeneous glycosylation of i-α1-ECD render it a promising material for structural studies as well, in order to elucidate the X-ray crystal structure of the human α1-ECD. 

Furthermore, we proceeded in assessing the conformation and antigenicity of i-α1-ECD and compared them to y-α1-ECD, using: a) binding of ligands, b) far-UV CD analysis, c) binding of mAbs and d) binding of autoantibodies from MG patients. ^125^I-α-BTX was found to have higher binding ability (about 1.5-fold increase) for y-α1-ECD than for i-α1-ECD (Figure **2A**), probably due to differences in the glycosylation motif conferred by the two different expression systems. Previous studies have shown the critical role of glycosylation of α1 in α-BTX binding [4,19]; it is therefore possible that the different glycosylation pattern of i-α1-ECD may have led to the observed 1.5-fold reduced ^125^I-α-BTX binding-ability compared to y-α1-ECD. Regarding binding of small cholinergic ligands, these seemed to bind similarly to the two molecules (Figure **2B**).

In order to further assess the native-like conformation of i-α1-ECD we performed far-UV CD studies (Figure **1B**). Interestingly, the α-helical content for the i-α1-ECD is 2-fold higher than that deduced for the y-α1-ECD [20], denoting the probably different folding of the α1-ECD between the two different expression systems. Moreover, taking into account the closer resemblance of the secondary structure composition of the i-α1-ECD to that deduced from the X-ray crystal structures of the homologous AChBP [21,22] and mouse α1-ECD [19], which in all cases is ~8% α-helix and ~45% β-sheet (compared to 8% and 40% for i-α1-ECD and 4.5% and 39.5% for y-α1-ECD [20]), we postulate that α1-ECD probably adopts a more native-like conformation when expressed in insect cells (i-α1-ECD) rather than in *yeast* (y-α1-ECD). 

Since the aforementioned ligand-binding studies provide information only on the conformation of the principal side of the ligand-binding site of muscle nAChR (contributed by the α1-ECD), and since the far-UV CD analysis is only indicative for the secondary structure composition the α1-ECDs under study, we proceeded to testing their binding ability to various anti-nAChR mAbs. Binding ability of these mAbs to the ECDs under study is indicative of their overall conformation, since the tested antibodies are partially or completely conformation-dependent and targeted towards various epitopes of α1-ECD. In addition, high antibody-binding efficiency is our main aim, since we wish to select the best anti-α1 auto-antibody binder for use in antigen-specific future therapies for MG [23]. ELISA experiments showed that all mAbs tested bound much better to i-α1-ECD than y-α1-ECD; in fact, mAb 64 which is an anti-α1 conformation-dependent antibody, in practice bound only to i-α1-ECD (Figure **3A**). These results indicated that i-α1-ECD presents a much improved antigenicity compared to y-α1-ECD, denoting in parallel a more native-like conformation. 

Finally, in order to further confirm the superiority of i-α1-ECD in terms of anti-α1 antibody binding, we proceeded in comparing the binding abilities of i-α1-ECD and y-α1-ECD to anti-nAChR autoantibodies present in five MG patients’ sera using RIA experiments. i-α1-ECD was found to be more effective than the y-α1-ECD in autoantibody binding, as well (Figure **3B**). Notably, serum 2 (low anti-nAChR-titer) and sera 4 and 5 (high anti-nAChR-titer) showed a similar profile of binding, as they saturated the available antigen at 3 and 5 microliters, while sera 1 and 3 (low anti-nAChR-titer) seemed to be still in a 'linear phase'. This could be due to differences in the percentages of the present anti-α1 antibodies in these sera and in their affinities to the α1-ECDs. Antibody titers were for the intact nAChR and not for the α1 subunit only; therefore it is likely that the differences between sera regarding their titers for the α1-ECDs were smaller than their differences for the intact nAChR and not necessarily proportional. For example, sera 2 and 3 with very similar anti-nAChR titers (16 and 15 nM, respectively) had very different binding efficiency (Figure **3B**). Nevertheless sera 4 and 5 with high anti-nAChR titers did bind much more efficiently than sera 1-3 (Figure 3B). Although sera 2, 4 and 5 (especially #4 and #5) seemed to approach saturation of the available antigen at high serum volumes, nevertheless the differences in binding between the two α1-ECDs (especially at low serum volumes) are apparent.

In conclusion, human α1-ECD expressed in lepidopteran insect cells appears to have improved expression yield, conformation and antigenicity than the corresponding molecule expressed in the yeast *P. pastoris*. This new recombinant protein (i-α1-ECD) seems to be the most promising human α1 molecule to be used as an effective autoantigen for the development of an antigen-specific therapeutic approach for MG [24].
